# Chitosan Inhibits the Rehabilitation of Damaged Microbes Induced by Photodynamic Inactivation

**DOI:** 10.3390/ijms19092598

**Published:** 2018-09-01

**Authors:** Ching-Hsuan Lin, Hsiung-Fei Chien, Ming-Hsuan Lin, Chueh-Pin Chen, Mandy Shen, Chin-Tin Chen

**Affiliations:** 1 Department of Biochemical Science and Technology, National Taiwan University, Taipei 106, Taiwan; chinghsuanlin@ntu.edu.tw (C.-H.L.); cc1103820@gmail.com (M.-H.L.); d00b22004@ntu.edu.tw (C.-P.C.); d03b22007@ntu.edu.tw (M.S.); 2 Division of Plastic Surgery, Department of Surgery, Taipei Medical University Hospital and College of Medicine, Taipei Medical University, Taipei 110, Taiwan; hfchien@h.tmu.edu.tw

**Keywords:** photodynamic treatment, microbial killing, cell wall

## Abstract

Previously, we showed that chitosan could augment the biocidal efficacy mediated by photodynamic treatment against *Staphylococcus aureus*, *Pseudomonas aeruginosa*, and *Candida albicans*. In this study, we showed that the antimicrobial action of chitosan in augmenting photodynamic inactivation (PDI) is related to the increase in cell surface destruction. The microbial cell surfaces exhibit severe irregular shapes after PDI in the presence of chitosan as demonstrated by transmitted electron microscopy. Furthermore, increases in the concentration or incubation time of chitosan significantly reduced the amounts of photosensitizer toluidine blue O required, indicating that chitosan could be an augmenting agent used in conjunction with PDI against *S. aureus*, *P. aeruginosa*, and *C. albicans*. A prolonged lag phase was found in microbial cells that survived to PDI, in which chitosan acted to completely eradicate the cells. Once the exponential log stage and cell rebuild began, their cellular functions from PDI-induced damage returned and the increased cytotoxic effect of chitosan disappeared. Together, our results suggest that chitosan can prevent the rehabilitation of PDI-surviving microbial cells, leading to increased biocidal efficacy.

## 1. Introduction

Infectious diseases caused by bacteria and fungi have become a serious problem in public health. Due to the emergence of resistance among human pathogens, conventional antimicrobial therapies have become less effective [1,2,3]. Indeed, many antibiotic and antifungal agent-resistant pathogens have been found that lead to critical issues in public health, such as methicillin-resistant *Staphylococcus aureus* (MRSA) [4], multidrug-resistant *Pseudomonas aeruginosa* [3,5,6], and pathogenic fungi [3,5,6]. Unlike the abundant range of antibiotics, only four classes of drugs (flucytosine, azoles, echinocandins, and polyenes) with relative toxicity to human cells are currently used to treat fungal infections [7]. Hence, development of a promising new drug or therapy against emerging infectious diseases or drug-resistant pathogens is urgently required.

Photodynamic therapy (PDT) is an approved therapeutic modality for the treatment of cancers and noncancerous diseases [8,9]. PDT activates nontoxic photosensitizers by a specific wavelength of visible light [10,11]. The activated photosensitizers react with O_2_ through the type I and type II photochemical reactions to generate reactive oxygen species (ROS), which directly cause oxidative damage to cells, microbes, or tissues, leading to cell death [9,12]. As summarized in numerous reviews, the use of the photodynamic principle to inactivate microbial cells, known as photodynamic inactivation (PDI), has been regarded as a new antimicrobial modality used for treating human infectious pathogens [12,13,14]. Specifically, several photosensitizers, such as acridine orange, chlorins, phthalocyanines, rose bengal (RB), methylene blue (MB), and toluidine blue O (TBO), have been studied in controlling infectious diseases [15,16,17,18]. Several clinical bacterial and fungal pathogens, including *Staphylococcus aureus*, *Pseudomonas aeruginosa*, *Klebsiella pneumonia*, *Escherichia coli*, *Candida albicans*, and many others can be efficiently controlled by PDI in vitro [12,13,19]. Presently, neither cytotoxicity nor DNA damage to keratinocytes was found in vitro in MB- or TBO-mediated PDI, which can cause significant killing of typical skin microbes [20,21,22]. Compared to bacteria, *Candida* are eukaryotic cells and higher doses of photosensitizers or light irradiation are required to effectively kill them, which might be harmful to human cells. Thus, the combination of PDI and an antimicrobial agent could be a promising treatment for infectious disease. 

Chitosan [poly-(*β*-1/4)-2-amino-2-deoxy-d-glucopyranose] is a linear polysaccharide that can be obtained by chitin deacetylation mediated by enzymatic hydrolysis of chitin deacetylase or NaOH alkaline conditions [23,24,25]. Due to its biodegradability, biocompatibility, and nontoxicity, chitosan has displayed its various important pharmacological applications in biomedical uses, dietary supplement food, agriculture, cosmetics industries, and in drug delivery [25,26,27,28]. A broad antimicrobial spectrum of chitosan has been reported to be effective against Gram-positive and Gram-negative bacteria and fungi [29,30,31]. In this regard, chitosan was used as an alternative antimicrobial treatment owing to its many exceptional advantages in both the clinical and environmental fields. It has been shown that degrees of chitosan deacetylation are correlated with its antimicrobial activity [27]. Although chitosan use against microorganisms shows a commercial potential, the exact mechanisms of its antimicrobial activity are still not fully understood. Previously, we showed that post-treatment with chitosan can significantly enhance the biocidal efficacy of PDI against different bacteria and *Candida* cells [32,33,34]. However, the mode of action of chitosan in augmenting the biocidal effect mediated by PDI is not clear. In this study, we further investigated the mechanism of chitosan in augmenting the PDI-mediated cytotoxicity against microbial cells. The effects of concentration and incubation time of chitosan in augmenting PDI efficacy were examined. Finally, we elucidated the effect of chitosan on the cell wall and growth rate in PDI surviving cells.

## 2. Results

### 2.1. Chitosan Treatment after PDI

**Figure 1:**
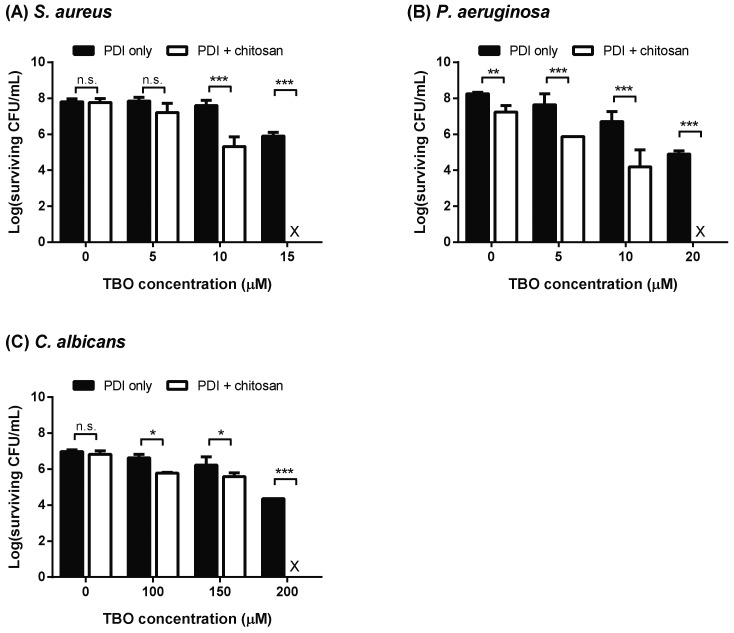
Chitosan augments the killing efficacy of photodynamic inactivation (PDI). Planktonic cells of (**A**) *Staphylococcus aureus*, (**B**) *Pseudomonas aeruginosa*, and (**C**) *Candida albicans* subjected to toluidine blue O (TBO)-mediated PDI under the light dose of 50 J cm^−2^. Following PDI, microbial cells were further treated with chitosan for 30 min. The concentrations of chitosan used for *S. aureus* and *P. aeruginosa* were 0.025% and 0.25%, respectively. For *C. albicans*, 0.25% chitosan was used post-incubation with PDI. The survival rate of each sample was then measured by plate count. At least three repeated experiments were performed to determine the surviving cells, expressed in log colony forming units (CFUs) mL^−1^. Each value is the mean from three independent experiments ± standard deviation (SD), X indicates the complete killing of cells, n.s. denotes no significance, * *p* < 0.05, ** *p* < 0.01, and *** *p* < 0.001.

To optimize the synergistic killing ability of PDI and chitosan for *S. aureus*, *P. aeruginosa*, and *C. albicans*, different concentrations of TBO were incubated with microbial cells for 30 min, washed with phosphate-buffered saline (PBS) and then irradiated with red light-emitting diode (LED) light (630 ± 5 nm). Microbial cells were further treated with or without chitosan following light irradiation. As expected, *C. albicans*, with a larger cell surface, required a 10-fold concentration of TBO to reduce two to three logs of viable cells compared to those of *S. aureus* and *P. aeruginosa* (Figure 1). Furthermore, chitosan addition to the microbial cells treated with PDI caused a complete eradication compared to those treated with PDI or chitosan alone. We found that two to three logs of cell killing induced by PDI was required for chitosan to further result in complete microbial cell death. The chitosan concentrations required for the complete killing of *S. aureus*, *P. aeruginosa* and *C. albicans* were 0.025%, 0.25% and 0.25%, respectively.

### 2.2. Morphologic Aspects Observed by TEM

**Figure 2:**
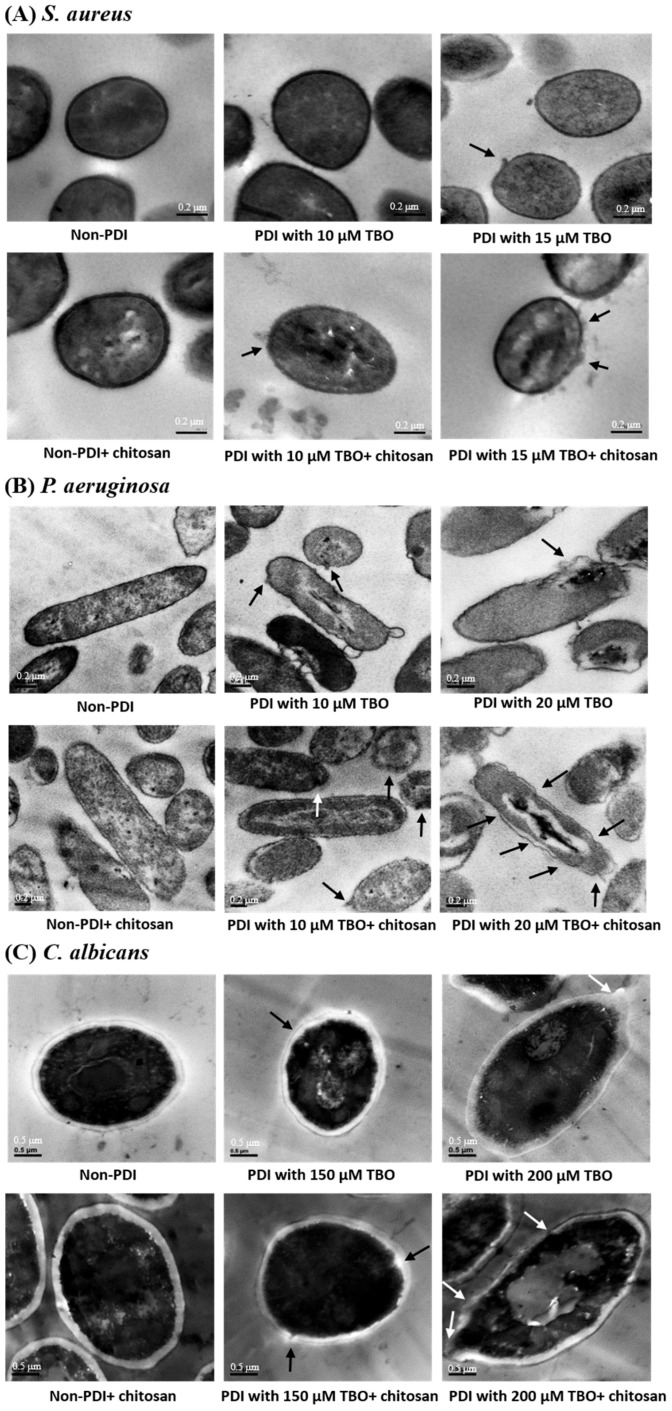
Transmission electron microscopy (TEM) showed that PDI combined with chitosan caused severe damage to the cell surface. Images of (**A**) *S. aureus*, (**B**) *P. aeruginosa*, and (**C**) *C. albicans* were taken after TBO-mediated PDI, chitosan, or combined treatment of PDI and chitosan. Arrows indicate irregular cell surfaces.

To observe microbial cell morphologies after treatments with PDI or chitosan alone or chitosan treatment following PDI, transmission electron microscopy (TEM) was used. As shown in Figure 2, there was either no or mild damage on the cell surfaces of *S. aureus*, *P. aeruginosa*, and *C. albicans* treated with PDI or chitosan alone, whereas post-incubation with chitosan after PDI caused a more severe corruption of cell surfaces, suggesting that chitosan might augment the damage to the cell surface induced by PDI.

### 2.3. Increasing the Incubation Time or Concentration of Chitosan in PDI-Induced Cytotoxicity

**Figure 3:**
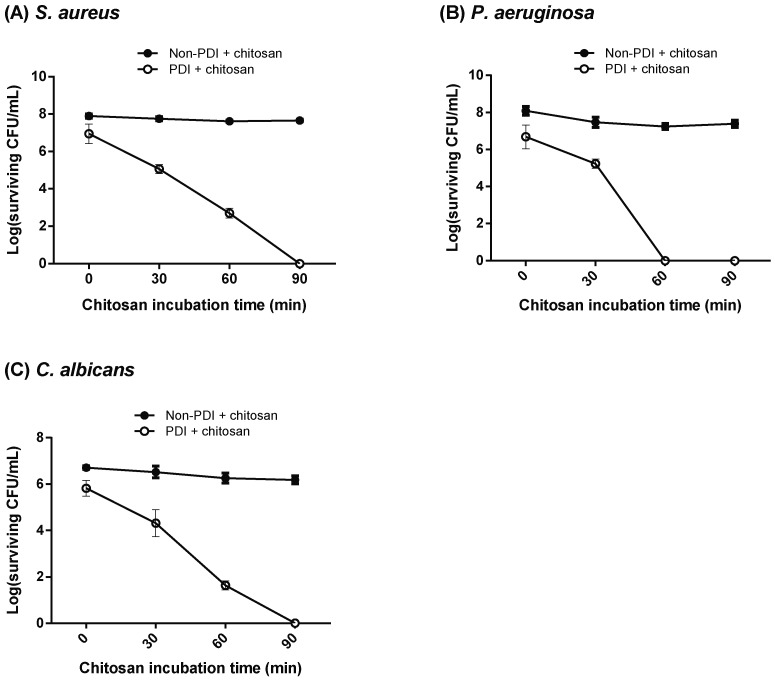
Increase in the chitosan incubation time dramatically enhanced the killing effect. The concentration of photosensitizer TBO used in PDI was 10 μM in (**A**) *S. aureus* and (**B**) *P. aeruginosa*. (**C**) For *C. albicans*, 150 μM of TBO was used for PDI. After light irradiation (50 J cm^−2^), chitosan was added and further incubated for different time periods. The concentrations of chitosan used for *S. aureus* and *P. aeruginosa* were 0.025% and 0.25%, respectively. For *C. albicans*, 0.25% chitosan was used for the post incubation after PDI. After each incubation period, an aliquot of microbial cells was removed for plate count. Each value is the mean obtained from three independent experiments ± SD.

As shown above, chitosan treatment following PDI exhibited an increased killing effect against microbial cells. We then further examined whether the increase in biocidal activity was correlated with the concentration or incubation time of chitosan. To this end, we performed low-dose PDI against bacteria and *C. albicans* by incubating with 10 μM and 150 μM of TBO, respectively. As shown in Figure 3A, 10 μM TBO-mediated PDI only resulted in a one log reduction in *S. aureus*. In the presence of 0.025% chitosan, PDI-induced cytotoxicity increased in an incubation time-dependent manner, in which complete cell death was found after incubation for 90 min. Similar results were also found in *P. aeruginosa* and *C. albicans* with the combination of PDI and 0.25% chitosan.

**Figure 4:**
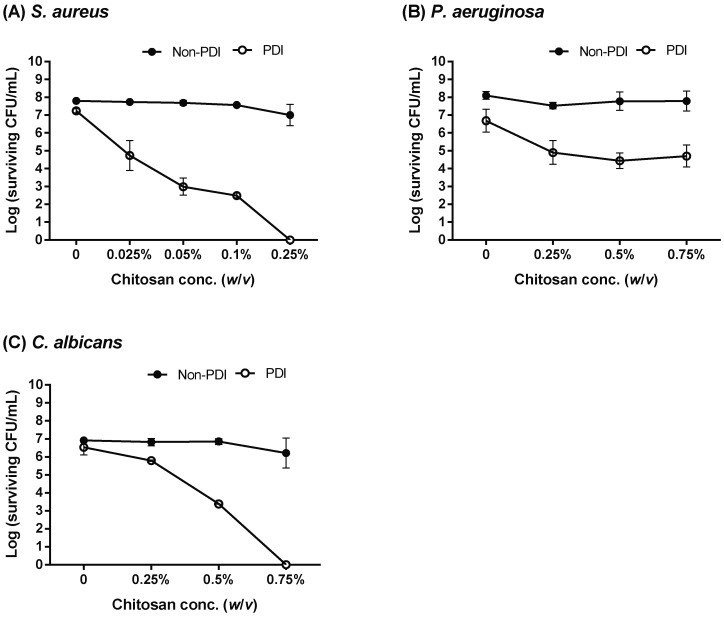
Increases in chitosan concentration could enhance the killing ability against microbial cells. Planktonic cells of (**A**) *S. aureus*, (**B**) *P. aeruginosa*, and (**C**) *C. albicans* subjected with TBO-mediated PDI were then incubated with different concentrations of chitosan for 30 min. For PDI, 10 μM TBO was used in *S. aureus* and *P. aeruginosa*. For *C. albicans*, 100 μM TBO was used. After light irradiation (50 J cm^−2^), cells were further incubated with different concentrations of chitosan as indicated for 30 min. Each value is the mean obtained from three independent experiments ± SD.

Furthermore, we assessed the increased level of cytotoxicity against microbial cells pre-treated with PDI under different concentrations of chitosan. As shown in Figure 4, less than a one-log reduction in viable cells was observed against bacteria (*S. aureus* and *P. aeruginosa*) and *C. albicans* in the presence of 10 μM and 100 μM TBO, respectively. However, in the presence of chitosan, increased cytotoxicity was found in PDI-treated cells in a concentration-dependent manner. Complete eradication of *S. aureus* and *C. albicans* was found by increasing the concentration of chitosan to 0.25% and 0.75%, respectively (Figure 4A,C, respectively). Notably, 0.75% chitosan did not completely eradicate the *P. aeruginosa* under the PDI condition of 10 μM TBO plus 50 J cm^−2^ light dose (Figure 4B). We found that 20 μM TBO-mediated PDI combined with 0.25% chitosan completely eradicated *P. aeruginosa* (Figure 1B), suggesting that the increased bactericidal effect of chitosan requires a certain level of damage induced by PDI. 

### 2.4. Prolonged Lag Phase in PDI-Surviving Cells

**Figure 5:**
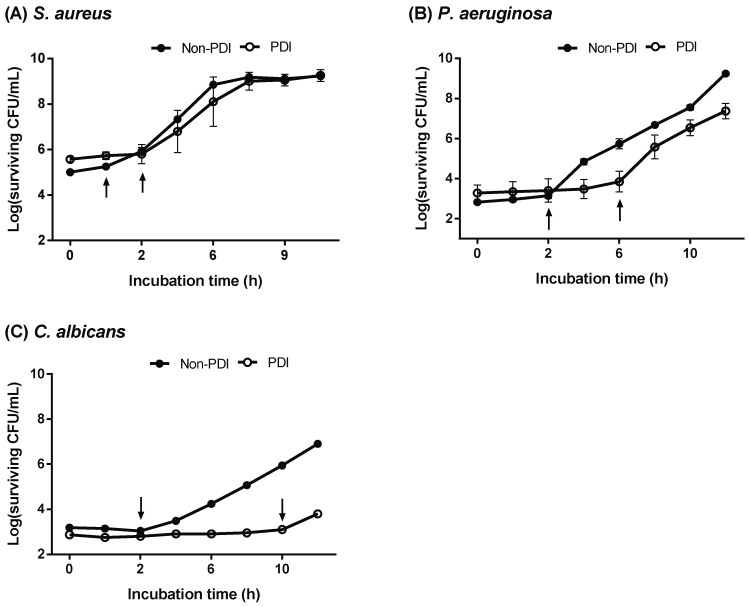
Prolonged recovery time in the PDI surviving cells. Cells of (**A**) *S. aureus*, (**B**) *P. aeruginosa*, and (**C**) *C. albicans* treated with or without PDI. For PDI, 15 μM and 20 μM TBO was incubated with *S. aureus* and *P. aeruginosa*, respectively. For *C. albicans*, 400 μM TBO was used for PDI. After light irradiation (50 J cm^−2^), cells were sub-cultured in a fresh culture medium. During the incubation, cells were collected every 2 h to perform plate count. Black arrows indicate the time that PDI or non-PDI treated cells enter the exponential growth phase.

As shown above, the augmented cytotoxicity of chitosan in PDI-treated cells was related to the damaged level induced by PDI. It was not clear whether the PDI-induced damage would affect the reproductive abilities of cells surviving PDI. We therefore further examined the growth curves of PDI surviving cells. To this end, microbial cells treated with PDI were introduced into a fresh culture medium. The growth condition of PDI-surviving cells was monitored by analyzing the number of viable cells at different incubation time points by counting the plate colony forming units (CFUs). As shown in Figure 5, a prolonged lag phase was found in the microbial cells that survived PDI compared to those without PDI treatment. The most predominant effect was in *C. albicans* treated with PDI, having a 10 h delay when entering the exponential (log) phase, whereas 2- and 6-hour delays were observed in PDI-treated *S. aureus* and *P. aeruginosa*, respectively. Although PDI-treated cells had a prolonged lag phase, the pattern of exponential growth was similar after leaving the lag phase, suggesting that the PDI-induced damage was temporary.

### 2.5. Chitosan Inhibits Recovery of Damaged Cells

**Figure 6:**
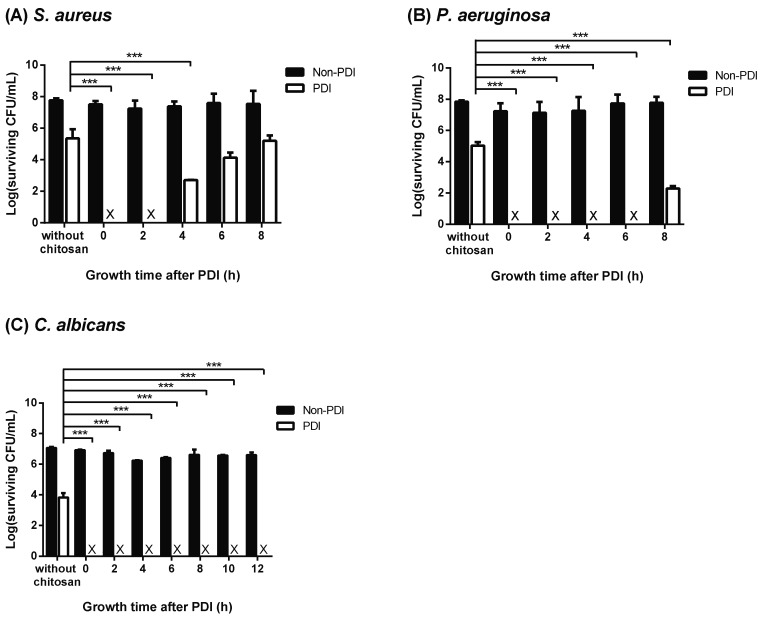
Chitosan inhibits the recovery of the PDI surviving cells. For PDI, 15 μM and 20 μM TBO was incubated with (**A**) *S. aureus* and (**B**) *P. aeruginosa*, respectively. (**C**) For *C. albicans*, 400 μM TBO was used for PDI. After light irradiation (50 J cm^−2^), cells were sub-cultured in a fresh liquid medium. Chitosan was then added at the time indicated and further incubated for 30 min. After incubation, cells were washed with phosphate-buffered saline (PBS) and plate counts were performed. Each value is the mean obtained from three independent experiments ± SD, X represents the complete killing of cells, and *** *p* < 0.001.

As shown above, cell wall damage induced by PDI was augmented by chitosan, which prolonged the lag phase of the surviving microbial cells. In this regard, we speculated that the augmented cytotoxicity mediated by chitosan might be related to the damage in the PDI-surviving cells. To examine the susceptibility to chitosan of surviving cells, chitosan was added at different time points after PDI. As shown in Figure 6, chitosan could completely eradicate the surviving cells of *S. aureus* in the first 2 h after PDI. After that, the increased cytotoxicity induced by chitosan gradually decreased and cell rehabilitation recovered. Compared to *S. aureus*, at least 6 and 10 h were required for the rehabilitation of damaged *P. aeruginosa* or *C. albicans*, respectively. Notably, 2, 6, and 10 h were required for PDI-treated *S. aureus*, *P. aeruginosa*, and *C. albicans* to enter the exponential (log) phase, respectively (Figure 5). These results indicate that chitosan might exert its augmented cytotoxicity during the recovery phase of PDI-surviving cells by inhibiting the repair of cell damage.

## 3. Discussion

Although the antimicrobial properties of chitosan have been documented in several reviews, the mechanism of chitosan in antimicrobial activity has not yet been fully elucidated [31,35]. Previously, we showed that post-treatment with chitosan could dramatically increase the antimicrobial effect of PDI against bacteria and *C. albicans* [32,34]. In this study, we used sub-lethal PDI to examine the mode of action of chitosan and its potentiating effect on TBO-mediated PDI. We demonstrated that chitosan enhances the damage on bacteria and *Candida albicans* surfaces after PDI (Figure 2). These results indicate that chitosan might exert its antimicrobial effect by interfering with the cell wall function and enhancing PDI-induced damage to the cell to further increase the biocidal efficacy. For clinical applications, photodynamic treatment is an ideal approach for treating superficial microbial infections. Considering microbial heterogeneity, a higher dose of PDI was required to result in sufficient killing efficacy against microbes. However, it is possible that a higher PDI dose might damage the healthy tissue cells in clinical application. In this regard, the present study further demonstrated the possibility of combining a lower dose of PDI with chitosan to efficiently function as a microbial killing agent while causing less damage to human tissues.

The antimicrobial actions of chitosan begin with its electrostatic interaction at the microbial cell surface [36]. As expected, a high positive charge of chitosan could lead to strong interactions with microbial cells. Therein, the antimicrobial activity of chitosan is related to its polycationic property, which is associated with its degree of deacetylation. In this study, the degree of deacetylation of chitosan used was about 90%, which indeed caused mild damage to the microbial cell surface as revealed by the TEM analysis (Figure 2). No significant cytotoxicity was observed in microbial cells treated with chitosan under different incubation times (Figure 3) or concentrations (Figure 4). We also found that there was no augmented cytotoxicity if the microbial cells were pre-treated with chitosan followed by photodynamic treatment. However, once the cell surfaces were damaged by PDI, the cells lost their protection imbued by the cell walls. Under this situation, chitosan further interacted and disrupted the unguarded cell membranes, leading to interference with the damage repair, ultimately leading to cell death. 

The surface structures of different microorganisms are complex and chemically heterogeneous. The cell wall in a bacterium or a fungus not only provides tensile strength for maintaining a definite shape but also protects microbes from environmental stress and immune evasion [37,38]. Thus, differences in microbial cell walls and the permeability barriers are responsible for the susceptibility to antimicrobial PDI. Compared to Gram-positive bacteria, most photosensitizers are less effective against Gram-negative bacteria [39,40]. The cell wall of Gram-negative bacteria consists of many-layered structures, including peptidoglycan, lipopolysaccharide, and lipoproteins, which might prevent the efficacy of a photosensitizer as well as chitosan [12,41]. This might explain why higher TBO concentrations were required to induce a 2- to 3-log reduction in the viability of *P. aeruginosa* and *C. albicans*, by which chitosan can further assist with complete cell killing. Moreover, the complex cell surface also explained why increased concentrations of chitosan in *P. aeruginosa* did not cause a strong bactericidal effect (Figure 4), unless a longer incubation time was used for complete cell killing (Figure 3).

In summary, we demonstrated that the augmented cytotoxicity mediated by chitosan after PDI is correlated to the increase in the chitosan concentration and incubation time. We found that a longer recovery time in the lag phase was required for the PDI surviving microbes to enter into the exponential growth stage. The addition of exogenous chitosan in the lag phase resulted in a significantly increased cytotoxicity. However, once the exponential log stage began and cells regained their physiological functions, the inhibitory effects of chitosan were mitigated. In the future, exploration of how chitosan enhances the effectiveness of PDI will provide useful guidelines for developing a better method to manage infectious diseases.

## 4. Materials and Methods

### 4.1. Strains and Reagents

The SC5314 *C. albicans* strain was grown in 50 mL yeast peptone dextrose (YPD) at 37 °C. *Staphylococcus aureus* and *Pseudomonas aeruginosa* were grown in tryptic soy broth (TSB) at 37 °C. The chitosan used in this study was purchased from Shin Era Technology (Taipei, Taiwan). As described previously, its molecular weight is ≈20 kDa and the degree of deacetylation of chitosan is ≈90% [32,34]. TBO was used as photosensitizer in the antimicrobial PDI assays and all other chemicals were obtained from Sigma-Aldrich Chemical Co. (St. Louis, MO, USA), unless otherwise stated.

### 4.2. PDI in Planktonic Microbial Cells

Overnight cultures of *C. albicans* harvested by centrifugation at 6000× *g* for 10 min were washed with PBS at pH 7.4 and re-suspended in PBS. The initial amounts of microbial were determined with OD_600_. One hundred microliters of *C. albicans* cells (10^7^ CFU mL^−1^) were transferred into a 96-well plate [34]. Preparations of *S. aureus* and *P. aeruginosa* for PDI followed the same procedure, but the final cell number was 10^8^ CFU mL^−1^ [32]. To prepare 2 mM TBO stock solution, TBO powder was dissolved with ddH_2_O and sterilized with a 0.22 μM filter. The concentration of TBO was calculated after determination with OD_630_. Next, 100 μL of TBO, dissolved in PBS with different concentrations, were added into each sample. Samples were incubated in a dark environment for 30 min at 25 °C with rotation speeds of 100 rpm. The samples were then centrifuged at 12,000× *g* for 1 min, washed with PBS, and re-suspended in 200 μL PBS. For light irradiation, microbial cells in microplate were placed at the bottom of a high-power red LED light source with a wavelength at 630 ± 5 nm and power density of 30 mJ cm^−2^. Light irradiation was performed at room temperature and the total light dose was 50 J cm^−2^. The total irradiation time was about 27.7 min. To determine the antimicrobial ability, PDI and non-PDI samples were diluted and plated on YPD and tryptic soy agar (TSA) for *C. albicans* and bacteria, respectively. Experiments were performed at least three times and all results are expressed as the mean ± SD. Survival assays were subjected to statistical analysis using student’s *t*-test.

### 4.3. Effect of Chitosan on TBO-Mediated PDI

To test the antimicrobial effect of chitosan on bacteria and *C. albicans*, chitosan (1% *w*/*v*) was dissolved in 1% acetic acid [32,34]. *S. aureus*, *P. aeruginosa*, and *C. albicans* were first treated with or without TBO-mediated photodynamic treatment. Chitosan incubation was performed immediately after light irradiation. For the dose-dependent study, a different concentration of chitosan was added and further incubated for 30 min. For the incubation time-dependent study, chitosan was added and further incubated for a different period of time. After the chitosan incubation, an aliquot of microbial cells was removed for plate count to test the survival rates of each microbe. Experiments were performed at least three times and all results are expressed as the mean ± SD. Survival assays were subjected to statistical analysis using Student’s *t*-test.

### 4.4. Survival Assay

Colony-forming units (CFUs) of *S. aureus*, *P. aeruginosa* and *C. albicans* suspensions, after PDI and non-PDI, with or without chitosan treatment, were enumerated as described previously [32,34]. Briefly, 10 μL of each sample with appropriate dilutions (from 10^−1^ to 10^−5^) were plated on YPD or TSA plates for *C. albicans* and bacteria, respectively. All the samples were incubated at 37 °C in a dark environment for 18 h. The samples having between 3 and 30 colonies were selected to count. The survival rate is expressed as follows: cell number (CFU mL^−1^) after PDI or PDI-chitosan treatment/initial sample cell number (CFU mL^−1^). Also, to evaluate the toxicity of TBO in the dark and minimize the intrinsic experimental error, each sample was monitored and the survival number of the non-illuminated samples was determined. Experiments were performed at least three times and all results are expressed as the mean ± SD. If the number of colonies was less than three colonies in the sample (without dilution), the treatment was regarded as completely killing of cells. Survival assays were subjected to statistical analysis using the two-tailed Student’s *t*-test and a *p*-value of <0.05 was considered significant.

### 4.5. Transmission Electron Microscopy (TEM)

Overnight cultures of microbial samples harvested by centrifugation at 6000× *g* for 10 min were washed with PBS (pH 7.4). Microbial cells treated with chitosan, PDI, or chitosan applied after PDI were pre-fixed with 3% glutaraldehyde for 2 h. Samples were washed with PBS three times and then fixed with 1% osmium tetroxide for 1.5 h, followed by dehydration for 5 min in a graded acetone series (30% and 50%), and 15 min in a graded acetone series (70%, 90%, and 100%), then incubated for 15 min each in acetone. A 3:1 (overnight), 1:1 (4 h), and 1:3 (4 h) mixture of acetone and Spurr (Electron Microscopy Sciences, Hatfield, PA, USA) were added in the dehydrated sample during the infiltration. Samples were embedded, trimming with Ultracut E (Leica, Wetzlar, Germany) and mounted on copper grids. Samples were observed using a transmission electron microscope (Hitachi Ltd., Tokyo, Japan). The control experiment was conducted in absence of any treatment.

### 4.6. Effects of Chitosan on Recovery of TBO-Mediated PDI Microbial Cells

For determining the growth curves, the remaining surviving cells of the PDI-treated and non-PDI-treated bacteria (*S. aureus* and *P. aeruginosa*) and *C. albicans* taken at different time points after PDI were re-inoculated on TSA or YPD medium, respectively. To examine the susceptibility to chitosan of surviving cells, chitosan was added into *S. aureus*, *P. aeruginosa*, or *C. albicans* at different time points after PDI. PDI-treated and non-PDI-treated bacteria and *C. albicans* were then incubated with chitosan for 30 min, then plated on the TSA and YPD plates, respectively, to determine their survival rates and chitosan inhibitory efficacy. Experiments were performed at least three times and all results are expressed as the mean ± SD. Survival assays were subjected to statistical analysis using the two-tailed Student’s *t*-test and a *p*-value of <0.05 was considered significant.

### 4.7. Statistical Analyses

All experiments were performed in three replicates with each replicate containing three technical repeats. The data are presented as the mean ± standard deviation. All statistical analyses were performed with Excel software (Redmond, WA, U.S.A.). Differences between two means were analyzed for significance using a two-tailed Student’s *t*-test with a 95% confidence interval. The *p*-value of <0.05 was considered statistically significant.

## 5. Conclusions

In this study, we demonstrated that PDI induces a prolonged lag phase in PDI-surviving cells, in which chitosan further severely damaged the microbial cell surfaces. The increased cytotoxicity of chitosan was not found once the cell growth entered the exponential log phase. This study gives a new insight on the mode of action of chitosan in increasing the antimicrobial efficacy, and might provide a better strategy for antimicrobial treatment.
